# Fire spreading across boundaries: The positive spillover of entrepreneurial passion to family and community domains

**DOI:** 10.3389/fpsyg.2022.952421

**Published:** 2022-09-02

**Authors:** Xiong-Hui Xiao, Hui Fu

**Affiliations:** School of Business, Sun Yat-sen University, Guangzhou, China

**Keywords:** entrepreneurial passion, positive spillover, work-life balance, work-family-community mesosystem, enrichment theory, entrepreneurial well-being, donation behavior, cluster entrepreneurship

## Abstract

Passion plays a crucial role in entrepreneurial activity, while its positive spillover to the family and community domains is scant. We proposed an integrated enrichment framework of “work-family-community” based on the literature in the field. Drawing upon the matching samples of entrepreneurs' individuals, families, and communities in the China Labor-force Dynamics Survey (CLDS) database, we identified a significant positive spillover effect into the family and community domains and explored the moderating role of the entrepreneur's perceived personal control. The empirical results indicate that entrepreneurs with higher passion experience higher subjective wellbeing related to family members and have a higher likelihood of engagement in prosocial behaviors. Perceived personal control positively moderates the spillover of passion to life and economic satisfaction. The spread of an entrepreneurial role model's peer effect and the contagion of entrepreneurial passion have a significant positive impact on entrepreneurial behavior in a cluster. Synthesizing our findings, we contribute to the literature concerning work-family enrichment, entrepreneurial passion, and the spillover-crossover model and offer important implications for entrepreneurs' role transition tension.

## Introduction

Entrepreneurs' role identities and emotional experiences differ from those of average salaried employees (Mathias and Williams, 2018). Entrepreneurs are required to manage conflicts between resource acquisition and role transitions, while facing high levels of uncertainty and heavy workloads (Farmer et al., 2011; Williamson et al., 2020; Stephan et al., in press). It is imperative to explore the positive transferring effects of entrepreneurs' resources from their work domain to other domains under role conflicts (Carlson et al., 2019). With this motivation, scholars advocate for the incorporation of entrepreneurial research into non-work domains, such as family (Greenhaus and Powell, 2006; Xu et al., 2020) and community (Anderson and Gaddefors, 2016; Samila and Sorenson, 2017), to assist entrepreneurs in developing more positive role images in their families and communities and addressing negative pressures and conflicts from the outside world. Along with positive emotions and identity characteristics during the entrepreneurial process, entrepreneurs can accumulate a number of resources in both the material and spiritual realms, thereby improving their economic and life satisfaction (Abreu et al., 2019; Wiklund et al., 2019; Stephan et al., in press). Due to the contagiousness of emotion and role identity, entrepreneurs may have a higher level of neighbor trust besides acting as role models in the same community (Barsade, 2002; Long and Yang, 2016; Barsade et al., 2018). Therefore, exploring the potential value of positive emotions and identity resources in entrepreneurship is conducive to the achievement of a higher level of work-life balance (Casper et al., 2018; Wayne et al., 2020; Rothbard et al., 2021).

Entrepreneurial passion refers to “intense positive feelings experienced by engagement in entrepreneurial activities associated with roles that are meaningful and salient to the self-identity of the entrepreneur” (Cardon et al., 2009; Zhao and Liu, in press). Prior studies investigating entrepreneurial passion mainly focused on the outcomes related to performance and mechanisms in the workplace (Li et al., 2020b; Newman et al., 2021; Adomako and Ahsan, 2022; Ahsan et al., 2022; Hu et al., 2022). Entrepreneurial passion plays crucial roles in stimulating entrepreneurial behavior (Feng and Chen, 2020; Li et al., 2020b), establishing entrepreneurial teams (Cardon et al., 2017b; Uy et al., 2021; Fu et al., 2022), obtaining funding (Chen et al., 2009a; Allison et al., 2022), motivating staff (Cardon, 2008; Zhu et al., 2019), triggering creativity (Davis et al., 2017; Luu and Nguyen, 2021; Murad et al., 2021), and achieving a venture's profit (Adomako et al., 2022), all of which have a direct impact on the survival and success of entrepreneurs and the business performance in the work domain (Drnovsek et al., 2016). However, the understanding of whether the indirect impact from entrepreneurial passion to non-work domains exists is still insufficient, particularly the impact of entrepreneurial passion on economic satisfaction, life satisfaction, neighbor trust, donation behavior, and cluster entrepreneurship in community. These important issues are compatible with the features of entrepreneurs which are embedded in the family and community domains (Tian and Zhang, 2018).

Furthermore, it is relevant to examine the impact of entrepreneurial passion on non-work domains in a sample from collectivist culture nations. Contextualization is of great importance to the theoretical construction of entrepreneurship research (Zahra, 2007; Welter, 2011). Due to the unique cultural value of China's context and the current stage of its economic transformation, entrepreneurial research in this context is still very limited (Yu et al., 2009; Van der Zwan et al., 2012; Sengupta et al., 2018). Still, national cultural characteristics are not only connected to the societal acknowledgment of entrepreneurial behavior, but also to the individual characteristics of entrepreneurship, particularly under the dichotomous division of individualism and collectivism (Hayton et al., 2002; Schmutzler et al., 2018). Compared to those in individualist nations, people in collectivist nations tend to embed themselves more in the social interaction network of extended families and other groups (Hofstede, 1984). For example, entrepreneurs in China would like to engage themselves in the non-work domains to achieve special social capital, namely “guanxi”, for opportunity exploitation and venture growth (Burt and Burzynska, 2017). People in collectivist nations may pay more attention to the value brought by work and less attention to the time or energy conflicts brought by work to other non-work roles (Shenkar and Ronen, 1987; Hsieh and Lin, 2010). They are more willing to see work as a contribution to their families rather than competition (Spector et al., 2007). Individuals in collectivist nations prefer to take work, family, and community as a mesosystem (Voydanoff, 2014), so as to achieve the multiple goals of physical and mental health, family harmony, social stability, world peace, and equality, which are values that are supported by the concept of human long-term development (Lin, 1938; Li and Liang, 2015). Thus, entrepreneurs in nations with a collectivist culture face the dilemma of work-non-work domain management. It is important to explore the specific issue of how to make the work domain contribute more to the family and community domains within entrepreneurial activities.

To address this issue, we investigate the positive impacts of entrepreneurial passion across multiple domains while considering positive emotion spillover and identity transition in work-life activities. In this study, we take entrepreneurial passion as a kind of psychological resource and examine whether its positive role can spill over to the family and community domains *via* the boundary cross and spillover mechanisms. This study focuses on the following two issues: (1) what are the positive outcomes of entrepreneurial passion spillover to the family and community domains, and (2) when is the positive enrichment be moderated relating to family members' subjective wellbeing and entrepreneurs' pro-social behavior in the community? Through a logistic study of 1894 entrepreneurs and 239 paired communities in China, we found that entrepreneurs with a higher level of entrepreneurial passion are apt to experience subjective wellbeing in life and economic dimensions. Entrepreneurial passion promotes a stronger perceived sense of trust in neighbors and a higher possibility of donation. As a moderator, an entrepreneur's perceived control significantly enhances the main effect between entrepreneurial passion and life satisfaction as well as economic satisfaction. The number of community entrepreneurs and the income gap together advance the spillover of entrepreneurial passion, which affects other members' entrepreneurial behavior in the same community.

We contribute to entrepreneurial passion research in the following three aspects. First, we introduce two novel functional domains into the research of entrepreneurial passion's indirect effect, namely the family and community domains, which extends the scope of current research. Second, we integrate enrichment theory (Greenhaus and Powell, 2006) into the spillover-crossover process (Bakker and Demerouti, 2013) to illustrate the positive effect of entrepreneurial passion from individual level to group level research. We assume that the perceived emotional resources between groups induced by entrepreneurial passion have a broader impact through the crossover between individuals when compared with the original state. These findings contribute to the understanding of how entrepreneurial passion can be treated as enrichment resources and operates in non-work domains. Third, we answer Cardon et al. (2013)'s call to explore the social antecedents and outcomes of entrepreneurial passion in future research. We answer this call by examining how entrepreneurial passion affects entrepreneurial wellbeing, as well as donation behavior and role modeling in the community. Under the positive spillover-crossover mechanism, we find that entrepreneurs' passion may predict their prosocial behaviors, especially when they have the opportunity to learn from others in the same community.

## Theoretical background and hypothesis development

It is crucial to present the theoretical background and framework logic underpinning this research before examining the spillover of entrepreneurial passion from the work domain to the family and community domains. First, the main topic of our research is entrepreneurial passion, which has been gaining more and more attention in business and management research during last two decades, especially in developing economies like China (Vallerand et al., 2003; Cardon et al., 2009; Chen et al., 2009a; Newman et al., 2021; Zhou et al., 2021; Zhao and Liu, in press). With regard to the conceptualization and measurement scales of entrepreneurial passion, there are four main ways defining entrepreneurial passion. Namely, entrepreneurs' passion for work (Baum and Locke, 2004), entrepreneurs' harmonious and obsessive passion (Vallerand et al., 2003; Ho and Pollack, 2014), perceived or observed entrepreneurial passion (Chen et al., 2009a; Li et al., 2017), and entrepreneurial passion toward different activities (Cardon et al., 2013), which correspond to the measurement of passion for work scale, dualistic model, displayed passion scale, and role-based model, respectively (Newman et al., 2021; Zhao and Liu, in press). The difference between the dualistic model and the role-based model is on how the activity is internalized into one's life, whether it is spontaneous or caused by internal pressure, external pressure, or an accidental event. The spontaneous type is harmonious passion, which is controlled by the actor. The other type is obsessive passion, which is the opposite (Vallerand, 2015; Vallerand and Houlfort, 2019; Fu et al., 2022). Nevertheless, the nature of entrepreneurial passion in the two dimensions of positive feeling and identity centrality are widely acknowledged (Zhao and Liu, in press). Newman et al. (2021) summarized the key outcomes of entrepreneurial passion in a recent review, which included entrepreneurs' attitudes and behavior (Li et al., 2020b), venture creation and funding outcomes, and others' attitudes or behavior (i.e., employees or entrepreneurial team members). Specifically, since entrepreneurial performance is the critical outcome variable of entrepreneurial research (Li et al., 2020a), the causal inference between entrepreneurial passion and entrepreneurial performance has been widely discussed (Ho and Pollack, 2014; Adomako and Ahsan, 2022; Allison et al., 2022). To expand the understanding of performance on attitudes and behavior in other domains (Cardon et al., 2017a), we focus on the positive spillover of entrepreneurial passion outside of the workspace while simultaneously accounting for the moderating effect of perceived personal control among entrepreneurs.

Second, the central framework theory of our research is enrichment theory, which specifically studies the beneficial effects of entrepreneurial passion. This implies that the experience in one role contributes to the quality of life in another role and generally has a positive impact on individuals, families, and organizations (Greenhaus and Powell, 2006). Both enrichment theory and conflict management theory (Greenhaus and Beutell, 1985) are key subjects in organizational behavior and human resource management that have attracted considerable research interest (Rothbard et al., 2021). Researchers who have written recent reviews proposed that both conflict and enrichment constructs should be included within the framework of work-life balance management and imply that balance may be assessed across borders (Casper et al., 2018; Vaziri et al., 2022). The enrichment theory of positive spillover is set in the context of balance management. Positive emotional resources are then treated as resources that could be used to achieve balance in the context of enrichment. In this way, the exploration of the positive spillover effect of entrepreneurial passion has become a pioneering attempt to expand boundary management research.

Third, the process mechanism created by the study of enrichment from entrepreneurial passion is based on spillover theory (Bolger et al., 1989; Grzywacz and Marks, 2000). Relevant studies have shown that there is a boundary between work and family; violation of that boundary occurs asymmetrically, with varying frequencies or degrees of boundary penetration, based on the needs of one domain (Clark, 2000; Jennings and Mcdougald, 2007; Kreiner et al., 2009). Hanson et al. (2006) defined positive spillover as “the transfer of characteristics from one domain to the other domain, resulting in similarities between the two domains.” Specifically, spillover can further be delineated into four types: affect, values, skills, and behaviors. For example, when individual needs are aligned with organizational resources, individuals' experiences at work can have a positive impact on the family domain (Chen et al., 2009b). Scholars further divide spillover into positive and negative spillover (Grzywacz and Marks, 2000), which diversifies the process mechanisms. In the study of close partners' effects on each other, Westman and Etzion (1995) referred to the interaction between different people in the same domain space as crossover. By extension, Bakker and Demerouti (2013) developed the spillover-crossover model, which shows how the experiences of one person in the work domain affect the experiences of other people in domains other than work. The research hypotheses are developed in the following 3 sections.

### The spillover of entrepreneurial passion to the family domain

Greenhaus and Powell (2006) defined work-family enrichment as “the extent to which experiences in one role improve the quality of life in the other role”, which has an instrumental path and an affective path in the positive effect process. The resources derived frome the entrepreneurial role, such as skills, knowledge, flexibility, material resources, and social-capital resources, can be transferred into driving factors of high performance in the family role (Greenhaus and Powell, 2006; Powell et al., 2019). Positive affect in one role promotes high performance in another of the same individual's roles (Greenhaus and Powell, 2006; Wayne et al., 2022). For entrepreneurial passion, positive affect is an important essential feature, which is accompanied by cognitive and behavioral manifestations of high value (Chen et al., 2009a). Entrepreneurial passion occurs in the work domain, accompanied by its two-dimensional nature, namely positive emotions and identity salience, could form a psycho-emotional resource that has a positive spillover from the work domain to the family domain (Bolger et al., 1989). The primary benefit of entrepreneurial passion penetrating the family domain is an improvement in entrepreneurs' subjective wellbeing, which represents an integral part of living a fulfilling and prosperous life and is intimately related to people's capacity to work, positive relationships maintain, and positive emotions experience (Ryff and Singer, 1998; Seligman, 2012). Along with the research on entrepreneurship, scholars recently proposed the concept of entrepreneurial wellbeing (Wiklund et al., 2019). Entrepreneurial well-being refers to the experience of satisfaction, positive affect, infrequent negative affect, and psychological functioning in relation to starting and developing an entrepreneurial venture (Wiklund et al., 2019), which is usually categorized into job satisfaction, life satisfaction, economic satisfaction, health satisfaction, and other dimensions (Abreu et al., 2019). Moreover, life satisfaction refers to the “quality of life when all things are considered” (Ferrer-i-Carbonell, 2013), while economic satisfaction in the family domain is mainly represented by the overall perception of the desirability of household income and its composition (Abreu et al., 2019). Participation in the family domain depends on the degree of people's good feelings and how they see themselves in the social world. Therefore, the positive affect and social identity of entrepreneurs have a positive effect on their role performance in the family domain.

In light of the spiritual and material resources triggered by entrepreneurial passion in the work domain, which can positively affect performance in the family domain, we propose that the spillover of entrepreneurial passion into the family domain contributes to entrepreneurial wellbeing (economic satisfaction and life satisfaction) in three aspects. First, work-family enrichment is conductive to balancing the spillover of resources (including psycho-emotional resources) from the work domain to the family domain, and enrichment impels people to be more effective and active as companions (Wayne et al., 2013). Second, regarding the framework of spillover effects (Bolger et al., 1989), individuals are more likely to invest resources from the work domain in building their families, and the investment of resources enhances the work-family enrichment effect and helps individuals identify as “good family members” (Carlson et al., 2006; Wayne et al., 2007). Third, according to the enrichment theory (Greenhaus and Powell, 2006), psychological resources related to positive emotions and identity salience, which are developed or nurtured in work roles, are conducive to forming the ability to acquire additional resources to cope with stress or conflict in family roles. Entrepreneurial passion, as an essentially positive affect and identity resource, contributes to wellbeing in the family domain, which can be measured through life satisfaction and economic satisfaction. Therefore, we propose the following hypotheses:

**Hypothesis 1a**: Entrepreneurial passion is positively related to life satisfaction from work-family enrichment. Entrepreneurs with higher passion experience greater life satisfaction due to the positive spillover of entrepreneurial passion to the family domain.
**Hypothesis 1b**: Entrepreneurial passion is positively related to economic satisfaction from work-family enrichment. Entrepreneurs with higher passion experience greater economic satisfaction due to the positive spillover of entrepreneurial passion to the family domain.

### The spillover of entrepreneurial passion to the community domain

Entrepreneurs are not single individuals in their work domains, they are also the constituent individuals in their community domains. In the process of entrepreneurial activities, due to the construction of entrepreneurs' social networks, the embedded community will not only be affected but also be reconstructed (Jennings et al., 2013). A community is composed of people who engage in social relationships and live in the same area, share a common history and values, participate in activities together (Phillips, 1993; Small and Supple, 2001) and is generally associated with entrepreneurship as a type of social capital (Samila and Sorenson, 2017). According to Anderson and Gaddefors (2016), entrepreneurship is not only an economic phenomenon but also a social one that is tightly linked to society at large. It stimulates changes in the community. For example, it may reduce crime and increase the sense of community security (K'nIfe and Haughton, 2013), bring novel ideas and a sense of empowerment to the local community (Vestrum, 2014), and regulate the relationship between community culture and socioeconomic development (Huggins and Thompson, 2014).

#### Entrepreneurial passion and neighbor trust

Due to the contagious characteristics of entrepreneurial passion (Cardon, 2008; Newman et al., 2021), it can be observed by employees and the team members in the same venture and perceived by individuals in the same community. Communities can be considered as a microsystem of non-work domains, where communities merge with work or family to form work-community mesosystems and community-family mesosystems (Bronfenbrenner, 1992; Voydanoff, 2014). The links and interactions between domains are the basic channels of spillover. Voydanoff (2001) proposed joint, moderating, and mediating roles between the work domain and the community domain, with psychological influences predominating. People in the same community with a high level of positive affect may predict harmony in the community (Aleem and Bano, 2014; Lomas, 2021), which, in the entrepreneurial context, may enhance the trust of the neighbors. The entrepreneur's instrumental and affective resources could facilitate the positive outcome of being a member in the community. The resources obtained from the work domain are essential for encouraging community involvement in specific activities such as donation and volunteer work. A positive view of the entrepreneur and trust from the community are the primary spillover effects of active participation from the work domain to the community domain (Wilson and Musick, 1997; Wilson, 2000). The main mechanisms that promote community development and harmony are influenced by socialization, peer comparison, and role modeling. Community identity, mutual trust, collective efficacy, and community norms are some of the main effects that spill over from the work domain to the community domain (Small and Supple, 2001). Notably, a community member's self-direction in decisions regarding his or her career and other areas is related to the society and organization to which he or she belongs. This is particularly clear in occupations with high social status (Wilson and Musick, 1997). Samila and Sorenson (2017) found that individuals with high levels of social trust in the community are more likely to choose entrepreneurship as a career, and those who join organizations closely related to the community have a higher rate of self-employment. Thus, we propose that:

**Hypothesis 2a**: Entrepreneurial passion is positively associated with work-community enrichment in trust among neighbors. Entrepreneurs with higher entrepreneurial passion experience greater trust in their neighbors as a result of the positive spillover of entrepreneurial passion into the community domain.

#### Entrepreneurial passion and donation behavior

From the perspective of enrichment, work-community enrichment enables psychological resources and social identity to flow into communities where entrepreneurs reside. For entrepreneurs, the pursuit of positive values and acknowledgment of one's identity will not only increase their commitment to the community, but it will also help cultivate a feeling of belonging among the community's members (Putnam, 2007; Greenaway et al., 2015). Statistical indicators from the Global Entrepreneurship Monitor Global Report show that an average of 65.8% of Chinese adults surveyed in 2016–2018 consider entrepreneurship a good career, and 73.7% consider successful entrepreneurs to have high social status (GEM, 2019), which explains the high vitality of entrepreneurship in China to a certain degree. The high degree of congruence between identity recognition from entrepreneurs themselves and from society makes it more likely for entrepreneurial passion to generate higher work-community enrichment in a collectivist society. Therefore, we propose that:

**Hypothesis 2b**: Entrepreneurial passion is positively associated with work-community enrichment in donation behavior. Entrepreneurs with higher entrepreneurial passion are more likely to engage in donation behaviors due to the positive spillover of entrepreneurial passion into the community domain.

#### Entrepreneurial passion and cluster entrepreneurship in the community

As with the embeddedness of entrepreneurs in communities, recent studies demonstrate that people who live in communities with a higher proportion of entrepreneurs are more likely to start up their own businesses (Bird and Wennberg, 2014; Nikolaev and Wood, 2018). On the one hand, the affective clues of entrepreneurial passion in enrichment theory can be contagious in the spillover-crossover process (Greenhaus and Powell, 2006; Bakker and Demerouti, 2013). The spillover of entrepreneurial passion from the work domain to the community domain results in crossover effects among community members with strong positive emotions and identity through emotional contagion mechanisms (Barsade, 2002; Cardon, 2008; Barsade et al., 2018) to which community members are subjected. On the other hand, the functional clues of entrepreneurial passion in enrichment theory can be learned by other members in the same community (Greenhaus and Powell, 2006), which then leads to a cluster of entrepreneurs in the community. The two-dimensional clues also allow entrepreneurial knowledge to spill over, which lowers the threshold and creates entrepreneurial clusters (Anderson and Gaddefors, 2016; Samila and Sorenson, 2017). The crossover of entrepreneurial passion leads to behavioral changes that are modified by social comparison mechanisms following emotional contagion (Festinger, 1954; Sullins, 1991). Specifically, the positive emotional experience and identity created by entrepreneurial passion spill over into the community domain. On this basis, community members will make social comparisons in entrepreneurial business performance, family living standards, entrepreneurial knowledge, and industry thresholds with those who have entrepreneurial passion so that they can make decisions that meet their own expectations in entrepreneurship and in the choice of career. This spillover-crossover effect is especially important in China, which is a collectivist society. Entrepreneurial passion spills over and crosses over into the community domain, affecting neighbors or relatives and making them want to become entrepreneurs. Based on the above discussion, we propose the following hypothesis:

**Hypothesis 3**: The greater the number and better the performance of community entrepreneurs, the more likely that entrepreneurial passion spillover will drive the development of entrepreneurial clusters in a community.

### The moderating effects of perceived personal control

#### Entrepreneurial passion and perceived personal control

The perceived personal control is considered as a belief in one's ability to exert control over a situation or event and is an important predictor of physical and mental health (Rothbaum et al., 1982; Lang and Heckhausen, 2001). We intend to explore the positive spillover effects of entrepreneurial passion within a theoretical framework of work-non-work enrichment. However, “changing the world is closely linked to changing oneself” (Rothbaum et al., 1982). To achieve a systematic balance in the work domain, family domain, and community domain, individuals must have a sense of control over their entire system, manage their involved domains and boundaries (Edwards and Rothbard, 2000), and then employ balanced resources to address conflicts between domains, the promotion of a domain, and the support of a domain (Duxbury et al., 1994; Bisconti and Bergeman, 1999; Wynn and Rao, 2020). According to the dualistic model, entrepreneurial passion and the personal sense of control have an intrinsic moderate connection. If the entrepreneur can better deal with the level of passion, he or she will experience harmonious passion, on the contrary, obsessive passion arises (Vallerand et al., 2003; Ho and Pollack, 2014). Prior literature indicates that harmonious passion plays a positive role in entrepreneurial performance, while obsessive passion would affect it negatively (Newman et al., 2021; Zhao and Liu, in press). Thus, it's relevant to investigate the impact of perceived personal control on the performance of entrepreneurial passion's spillovers into other domains.

Under the framework of enrichment theory, the perceived personal control may not only mitigate work-non-work conflict (Thomas and Ganster, 1995; Kelly and Moen, 2007), but also enhance the individual's enrichment experience (Carlson et al., 2011). Since the entrepreneur's high perceived personal control over work issues can be viewed as a resource (Kossek et al., 2006), the flexibility provided by the entrepreneur's vocational identity may strengthen the entrepreneur's ties to family and community (Kirkwood and Tootell, 2008; Kossek and Michel, 2011). As entrepreneurs shift roles among work, family, community, and other domains, they necessarily need to draw boundaries (Ashforth et al., 2000). Appropriately allocating time to multiple roles and avoiding conflict require a better sense of control to deal with the conflicts associated with role transitions (Wynn and Rao, 2020).

Specifically, the most indirect impact of entrepreneurial passion's spillover is into the family. Work-family enrichment can improve satisfaction when crossing domain borders (Daniel and Sonnentag, 2016). In accordance with the affective path of enrichment theory, entrepreneurs with passion would experience lower levels of stress (Baron et al., 2016), morality (Toivanen et al., 2016), and a higher level of health capital (Torrès and Thurik, 2019) than paid employees. The relationship between entrepreneurial passion and subjective wellbeing (including life satisfaction and economic satisfaction) would be strengthened under the role of perceived personal control due to the harmonious passion process. Another aspect of entrepreneurial passion's spillover is into the community. Work-community enrichment can consolidate the identity salience of entrepreneurs when crossing domain borders (Voydanoff, 2001). According to the functional cognitive path of enrichment theory, entrepreneurs tend to embed themselves in the communities to get social support (Jack and Anderson, 2002; Wang and Altinay, 2012; Wigren-Kristoferson et al., 2022). In the process of entrepreneurs' engagement in social activities (Zimmeran and Rappaport, 1988), maintaining a better neighbor identity (Greenaway et al., 2015), and exhibiting more prosocial behavior (Long and Yang, 2016), a sense of perceived control enables them to switch among their multiple identities more freely (Chasserio et al., 2014; Oo et al., 2019). If an entrepreneur has a strong sense of perceived personal control, he or she would gain an augmented inclination for neighbor trust and donation behavior. The following hypotheses are proposed based on the prior discussion.

**Hypothesis 4**: The positive spillover effects of entrepreneurial passion are positively moderated by perceived personal control. This includes the following hypotheses.
**Hypothesis 4a**: Perceived personal control positively moderates the positive spillover effects of entrepreneurial passion into the family domain in terms of life satisfaction.
**Hypothesis 4b**: Perceived personal control positively moderates the positive spillover effects of entrepreneurial passion into the family domain in terms of economic satisfaction.
**Hypothesis 4c**: Perceived personal control positively moderates the positive spillover effects of entrepreneurial passion into the community domain in terms of neighborhood trust.
**Hypothesis 4d**: Perceived personal control positively moderates the positive spillover effects of entrepreneurial passion into the community domain in terms of donation behavior.

**Figure 1:**
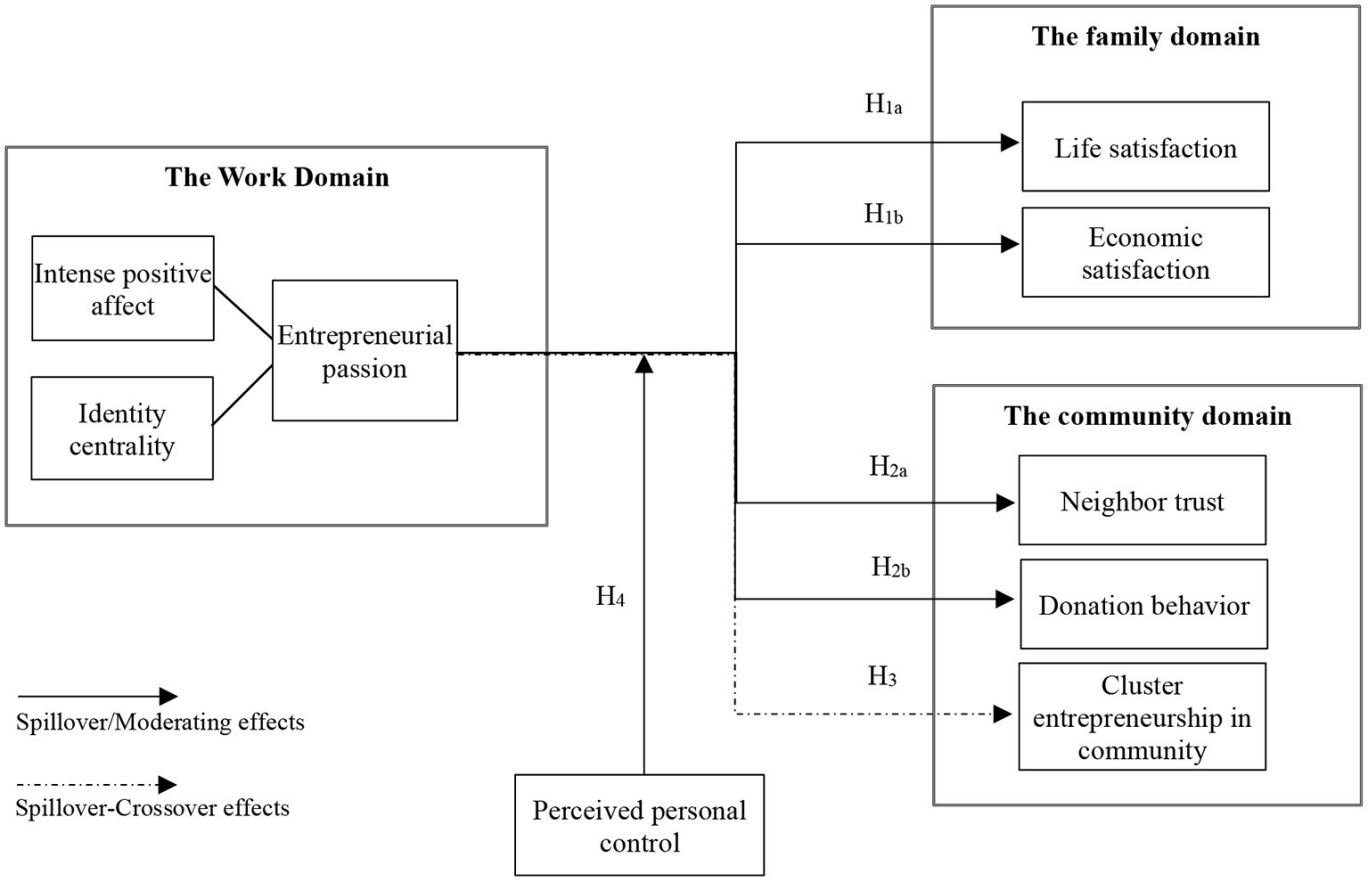
The theoretical model of entrepreneurial passion spillover study.

Based on the inferences and hypotheses of the above three parts, the overall theoretical model of this study is as shown in Figure 1.

## Methods

### Data and sample

The data used in this study are drawn from the China Labor-force Dynamics Survey (CLDS), a nationwide large-scale interdisciplinary follow-up survey focusing on the labor force in rural and urban villages in China; the purpose of the CLDS is to study the current situation and changes in the levels of education, employment, occupational mobility, occupational satisfaction, and happiness of the labor force. The CLDS has established a comprehensive database with tracking and cross-sectional data for three levels of the labor force; the study subjects are the individual, family, and community. The CLDS database possesses the following advantages: First, the sample is nationally representative, encompassing 29 Chinese provinces and cities. The subjects of the survey are employees of sample households and located communities. Second, the database includes longitudinal monitoring data, which is more suited to assessing entrepreneurs' families, communities, and emotional states. The CLDS has over 21,000 samples, including over 2,000 samples from entrepreneurs.

The key variable of this study, the spillover effect test of entrepreneurial passion, relies on whether participants trust their surroundings. We use the latest data in CLDS 2016, which includes information on workers' trust degrees of different groups and their emotional states toward family. Moreover, the spillover-crossover effect test of the number of entrepreneurs and industry choices of the same community members uses longitudinal tracking data after combining data from 2014 and 2016. The data of CLDS 2016 contains 21,086 individuals from 14,226 households and 401 communities, including entrepreneurs and non-entrepreneurs. To ensure the rationality and validity of the data samples, strict screening is carried out according to the following procedures: (1) We merge the data from individual level, family level, and community level. Main continuous variables are subjected to tail reduction at the 1% level to limit the influence of outliers; (2) The individual's current occupational status is used to identify the entrepreneur sample, and the sample whose occupational status is self-employed or employer is retained. In total 2,076 samples of entrepreneurs are obtained in this stage; (3) Samples with missing values of key variables are removed for accuracy. After these steps, the number of effective samples of entrepreneurs is 1,894 and that of the matching tracking community is 239.

### Variables

#### Dependent variables

##### Life satisfaction

In line with our theoretical principles and prior studies (Binder and Coad, 2013; Abreu et al., 2019), life satisfaction is evaluated by the entrepreneur's overall judgment on living standards, which shows how happy one's family life is at the material and spiritual levels. According to the CLDS question items, the respondent selects an option from “the unhappiest (Score 1)” to “the happiest (Score 5).”

##### Economic satisfaction

To measure the entrepreneur's economic satisfaction based on the material dimension of the family, he or she provides an overall judgment of the situation of the family's income situation, which is used to determine how happy one's family life is at the economic level (Carter, 2011; Abreu et al., 2019). According to the CLDS survey question items, the respondent selects an option from “the unhappiest (Score 1)” to “the happiest (Score 5).”

##### Neighbor trust

Neighbor trust, which is evaluated by the entrepreneur's trust in the relationship between members of the community (including rural villages), is a positive perception of the community (Kwon et al., 2013; Xiong et al., 2018). This study referred to the evaluation of neighbor trust in CLDS and scored entrepreneurs and communities from “completely untrustworthy (Score 1)” to “completely trustworthy (Score 5).”

##### Donation behavior

According to prior studies, entrepreneurs' donation behavior, which is considered a community-friendly action, is evaluated based on whether they have donated to the community (Long and Yang, 2016; Xu et al., in press). The CLDS is designed to gather information on whether a person has made charitable gifts or not, with donations being coded as 2 and no donations as 1.

##### Cluster entrepreneurship in community

Cluster entrepreneurship in the community is mainly focused on the number of cluster entrepreneurs and industry choices in the community, which belong to the outcome of crossover expansion analysis after spillover. According to Nikolaev and Wood (2018), the contagion effects of cluster entrepreneurship in the community are analyzed by the following three indicators: the growth rate of the entrepreneurial population, the ratio in the overall population and industry clusters. They are calculated in the following manner: the growth rate of entrepreneurial population = (2016–2014)/2014 × 100%; Growth rate of the ratio in overall population = (proportion of community entrepreneurial population in 2016-proportion of community entrepreneurial population in 2014)/proportion of community entrepreneurial population in 2014 × 100%; Growth rate of industry cluster = (industry convergence in 2016 - that in 2014)/industry convergence in 2014 × 100%. More specifically, the industry convergence rate equals the entrepreneurial population in the top three industries divided by the population of the community, and its ranking is based on the number of entrepreneurs in that industry.

#### Independent variables

##### Entrepreneurial passion

Referring to the measurement of an entrepreneur's passion by Cardon et al. (2013); Ho and Pollack (2014), combined with the questionnaire items designed by CLDS, this study comprehensively characterized entrepreneurial passion from four dimensions: sense of job value, respect, hard work, and mental and physical exhaustion. Specifically, four proxy measures of the expression of entrepreneurial passion are used, such as “I feel that I have created a lot of value,” “even if I need to rest for illness or other reasons, I will still work hard to complete my tasks,” “I am satisfied with the respect that others give to my work,” and “It has made me feel exhausted, physically and mentally.” Our measure captures both harmonious and obsessive aspects of entrepreneurial passion. Compared to the dualistic model of passion used in Ho and Pollack (2014), the consistency of proxy expression is acceptable when dealing with large-scale national data (Nikolaev et al., 2020).

##### Entrepreneurial income disparity

Entrepreneurial income disparity includes the difference between entrepreneurs' average income and per capita income, which is mainly determined by the difference between the entrepreneur's average income and the whole community's average income. Entrepreneurs' average income is derived from the operational income variable in CLDS, while the community's average income is derived from the average income of community owners, including operational income and wage. The income difference is used to indirectly evaluate entrepreneurs' business performance, which is used to correct the benchmark for decision-making in the social comparison of entrepreneurial passion spillover-crossover (Hagerty, 2000; Cheung and Lucas, 2016).

#### Moderating variable

##### Perceived personal control

Perceived personal control is measured by how much the entrepreneur is in control of work, family, and community (Lang and Heckhausen, 2001; Greenaway et al., 2015). To determine an individual's life control level, he or she responds by choosing an option from “no choice at all (Score 1)” to “a lot of choice (Score 10).”

#### Control variables

On the path of positive spillover of entrepreneurial passion, the entrepreneurs' marriage and family income affects their feelings about wellbeing (Abreu et al., 2019), and their monthly working hours are related to their family outcomes, while income, education level, and age affect their levels of donation and trust in neighbors. Therefore, the control variables include gender, age, marriage, education, family income, and working hours per month.

### Analytical strategy

We conduct a separate module analysis between entrepreneurial passion's spillover and crossover into the family and community domains. For the spillover, life satisfaction, economic satisfaction, neighbor trust, and donation behavior are ordered multiclassification variables. The following logit model is constructed to test hypothesis H1a, hypothesis H1b, hypothesis H2a, hypothesis H2b, and hypothesis H4 (including hypothesis H4a, hypothesis H4b, hypothesis H4c, and hypothesis H4d).


(1) $$\begin{array}{l} oLogit\left(\frac{\sum_{k=1}^{j}P(y_{i}=k|X_{i})}{1-\sum_{k=1}^{j}P(y_{i}=k|X_{i})}\right)=\beta_{j}+\beta_{1}^{T}Etrepassion_{i}\\ +\beta_{2}^{T}Lifecontrol_{i}+\beta_{3}^{T}\sum_{i}Control_{i}+\varepsilon \end{array}$$

For the crossover, the following model is constructed to test hypothesis H3:


(2) $$\begin{array}{l} OLS\left(\sum \left(Y_{i}-{\overset{\text{O}}{\beta }}_{0}-{\overset{\text{O}}{\beta }}_{1}X_{i}\right)\right)=\beta_{0}+\beta_{1}Etretotalincome\\ \;+\beta_{2}\sum_{i}Control_{i}+\varepsilon \end{array}$$

where *Etrepassion* represents the entrepreneurial passion of entrepreneurs, *Etretotalincome* represents the difference between the average income and per capita income of entrepreneurs, *Lifecontrol* is the perceived personal control of moderating variables, ε are all control variables, and β_*j*_ are residual items and initial values at different levels of dependent variables *j*.

## Results

### Non-response bias

The sample screening process in this paper excluded 182 samples with missing values, which may bias the study results. In order to determine whether non-response bias was an issue in this study, we used the approach recommended by Armstrong and Overton (1977). A *t*-test was conducted to compare the gender and age of the excluded 182 samples with the retained 1,894 samples. The results showed that there were no significant differences (*P* < 0.05) between the two datasets in terms of gender and age. Therefore, this paper excludes the potential threat of non-response bias.

### Common method variance

Despite the fact that this study used data from three different sources—individuals, households, and communities—common method variance may still be a concern because the data are cross-sectional (Podsakoff et al., 2012). We checked the current data for potential issues with common method bias using Harman's single factor test. If one component accounts for more than 50% of the total variance, there is a strong risk of common method bias (Podsakoff et al., 2003, 2012). The results of exploratory factor analysis revealed that the study's primary four factors explained 62.829 percent of the cumulative variance, but the largest factor only explained 27.177 percent of the variance, falling short of the threshold value of 50 percent, indicating that this paper is free of common method bias issues.

### Descriptive statistics

The descriptive statistical results of this study are shown in Table 1 below. Table 1 shows that the average level of entrepreneurial passion of entrepreneurs is 12.4, which is at the medium level in the range of 0–20. The average value of donation behavior is 0.26, indicating that 26% of entrepreneurs have donated. The average gender is 1.38, indicating that there are more male entrepreneurs in the statistical sample, with an average age of 42.58 years. From the correlation coefficient result, the correlation coefficients between entrepreneurial passion and dependent variables such as life satisfaction, economic satisfaction, neighbor trust, and donation behavior are 0.30 (*P* < 0.01), 0.26 (*P* < 0.01), 0.13 (*P* < 0.01) and 0.08 (*P* < 0.01), indicating that entrepreneurial passion is significantly positively correlated with entrepreneurs' life satisfaction, economic satisfaction, neighbor trust, and donation behavior.

**Table 1:** Descriptive statistics and correlation analysis.

**Variable name**	**Mean value**	**Standard deviation**	**1**	**2**	**3**	**4**	**5**	**6**	**7**	**8**	**9**	**10**	**11**	**12**
1. Entrepreneurial passion	12.4	2.33	1											
2. Life satisfaction	3.71	0.96	0.30***	1										
3. Economic satisfaction	3.25	1.08	0.26***	0.64***	1									
4. Neighbor trust	3.7	0.79	0.13***	0.20***	0.18***	1								
5. Donation behavior	0.26	0.44	0.08***	0.04*	0.06**	−0.01	1							
6. Perceived personal control	6.95	2.21	0.23***	0.30***	0.26***	0.06***	0.03	1						
7. Monthly working hours	216.12	115.45	−0.04	−0.02	−0.02	−0.03	−0.03	−0.06***	1					
8. Gender	1.38	0.49	−0.03	0.01	0.03	−0.08***	0.03	−0.04*	0.03	1				
9. Age	42.58	10.24	−0.07***	−0.02	−0.02	0.11***	−0.02	0.02	−0.07***	−0.05**	1			
10. Marital status	2.05	0.62	−0.04*	−0.04*	−0.07***	−0.03	−0.02	−0.01	0.01	0.09***	0.22***	1		
11. Education level	3.64	1.98	0.16***	0.05**	0.10***	−0.07***	0.12***	0.08***	−0.03	−0.04	−0.31***	−0.11***	1	
12. Family income	77630.82	76536.26	0.12***	0.16***	0.24***	−0.04*	0.18***	0.11***	0.02	0.01	−0.09***	−0.08***	0.28***	1

*P < 0.1, **P < 0.05, ***P < 0.01

### Hypothesis testing

#### Tests of the main spillover effect

Table 2 shows the hypothesized test result of the relationship between entrepreneurial passion and life satisfaction, economic satisfaction, neighbor trust, and donation behavior. Model 1 and Model 2 are used to test the spillover of entrepreneurial passion into the family domain, testing the relationship between the positive spillover of entrepreneurial passion and life satisfaction and economic satisfaction, respectively; and Model 3 and Model 4 are used to test the spillover of entrepreneurial passion into the community domain, testing the relationship between the positive spillover of entrepreneurial passion and neighbor trust and donation behavior, respectively.

**Table 2:** The spillover test of entrepreneurial passion with life satisfaction, economic satisfaction, neighbor trust, and donation behaviour.

	**Model 1**	**Model 2**	**Model 3**	**Model 4**
Gender	0.099	0.178**	−0.247***	0.162
	(0.089)	(0.087)	(0.091)	(0.111)
Age	−0.002	0.004	0.018***	0.006
	(0.005)	(0.004)	(0.005)	(0.006)
Marital status	−0.096	−0.169**	−0.139*	−0.020
	(0.073)	(0.071)	(0.073)	(0.091)
Education level	−0.052**	−0.004	−0.071***	0.082***
	(0.024)	(0.023)	(0.024)	(0.028)
Family income	0.000***	0.000***	−0.000*	0.000***
	(0.000)	(0.000)	(0.000)	(0.000)
Monthly working hours	0.000	−0.000	−0.000	−0.000
	(0.000)	(0.000)	(0.000)	(0.000)
Entrepreneurial passion	0.217***	0.176***	0.111***	0.052**
	(0.020)	(0.020)	(0.020)	(0.024)
Perceived personal control	0.226***	0.184***	0.036*	−0.005
	(0.021)	(0.020)	(0.021)	(0.025)
N	1,894	1,894	1,894	1,894
Log likelihood	−2,371.3009	−2,617.4721	−2,151.436	−1,048.7902
chi2	332.547	323.888	83.487	76.416

*P < 0.1, **P < 0.05, ***P < 0.01; Inside parentheses is the standard error of a coefficient estimate.

Table 2 shows that the positive spillover of entrepreneurial passion to the family domain is significant. The results of Model 1 show that there is a significant positive correlation between entrepreneurial passion and life satisfaction (beta = 0.217, *P* < 0.01). Entrepreneurs with high entrepreneurial passion experience higher life satisfaction, and hypothesis H1a is verified. The results of Model 2 show that there is a significant positive correlation between entrepreneurial passion and economic satisfaction (beta = 0.176, *P* < 0.01). Entrepreneurs with high entrepreneurial passion experience higher economic satisfaction. Hypothesis H1b is verified. Furthermore, from the results of Model 3 and Model 4, we can see that the spillover of entrepreneurial passion to the community domain is significant, and there is a significant positive correlation between entrepreneurial passion and neighbor trust and donation behavior (beta = 0.111, *P* < 0.01; beta = 0.052, *P* < 0.05), indicating that after controlling for other variables, entrepreneurs with high entrepreneurial passion perceive stronger neighbor trust and have a greater likelihood of donation behavior. Hypothesis H2a and hypothesis H2b are verified.

#### Tests of the moderating effect

We tested the moderating effects of entrepreneurs' perceived control on life satisfaction, economic satisfaction, neighbor trust and donation behavior. The specific tests are shown in Table 3:

**Table 3:** The moderating effect test of perceived personal control.

	**Model 5**	**Model 6**	**Model 7**	**Model 8**
Gender	0.098	0.178**	0.162	−0.247***
	(0.089)	(0.087)	(0.111)	(0.091)
Age	−0.002	0.004	0.006	0.018***
	(0.005)	(0.004)	(0.006)	(0.005)
Marital status	−0.103	−0.179**	−0.019	−0.140*
	(0.073)	(0.071)	(0.092)	(0.074)
Education level	−0.054**	−0.006	0.082***	−0.071***
	(0.024)	(0.023)	(0.028)	(0.024)
Family income	0.000***	0.000***	0.000***	−0.000*
	(0.000)	(0.000)	(0.000)	(0.000)
Monthly working hours	0.000	0.000	0.000	0.000
	(0.000)	(0.000)	(0.000)	(0.000)
Entrepreneurial passion	0.107*	0.056	0.060	0.097
	(0.059)	(0.056)	(0.072)	(0.059)
Perceived personal control	0.036	−0.026	0.01000	0.013
	(0.098)	(0.093)	(0.120)	(0.097)
Perceived personal control × Entrepreneurial passion	0.016**	0.017**	−0.001	0.002
	(0.008)	(0.008)	(0.010)	(0.008)
N	1,894	1,894	1,894	1,894
Log likelihood	−2,369.3373	−2,614.8165	−1,048.7828	−2,151.4055
chi2	336.47400	329.19900	76.43100	83.54800

*P < 0.1, **P < 0.05, ***P < 0.01; Inside parentheses is the standard error of a coefficient estimate.

**Figure 2:**
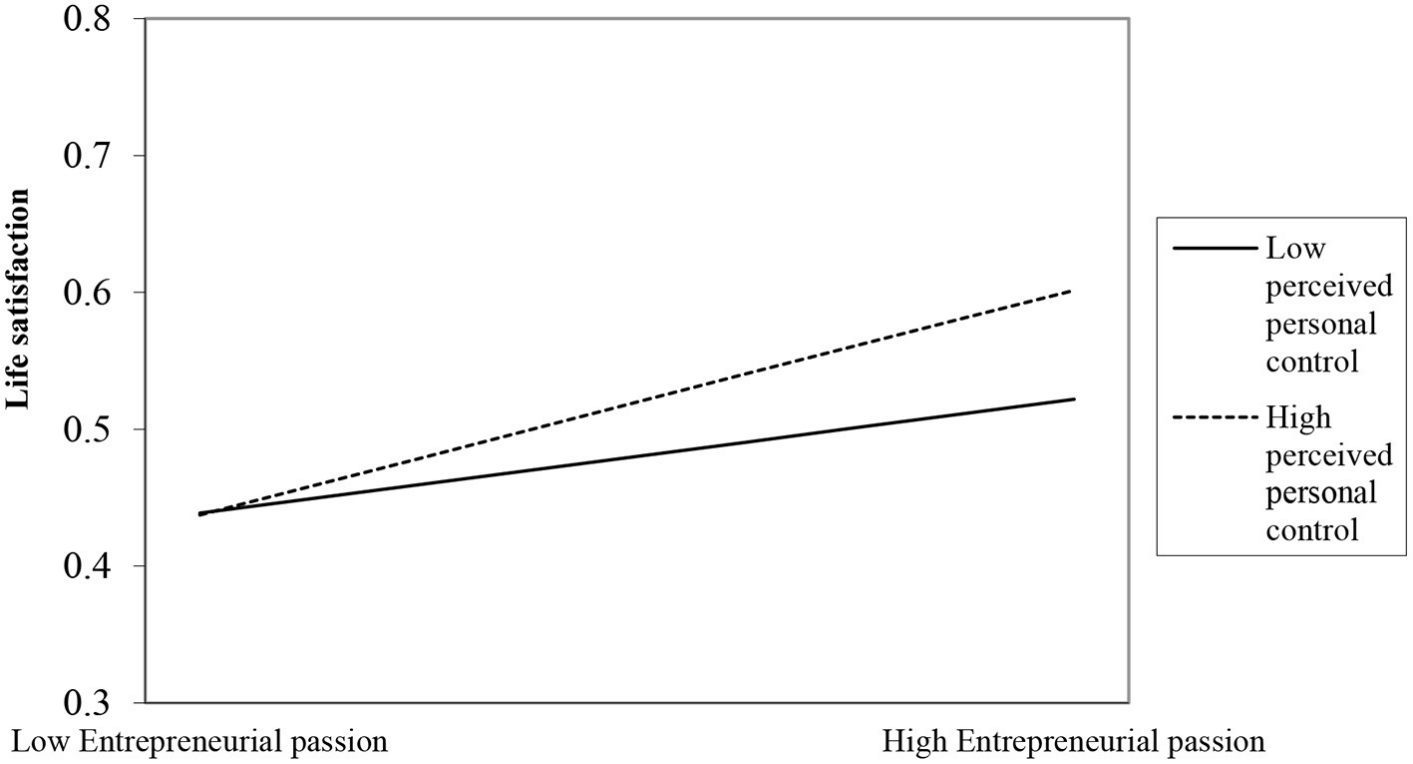
The moderating effect of perceived personal control on entrepreneurial passion and life satisfaction.

**Figure 3:**
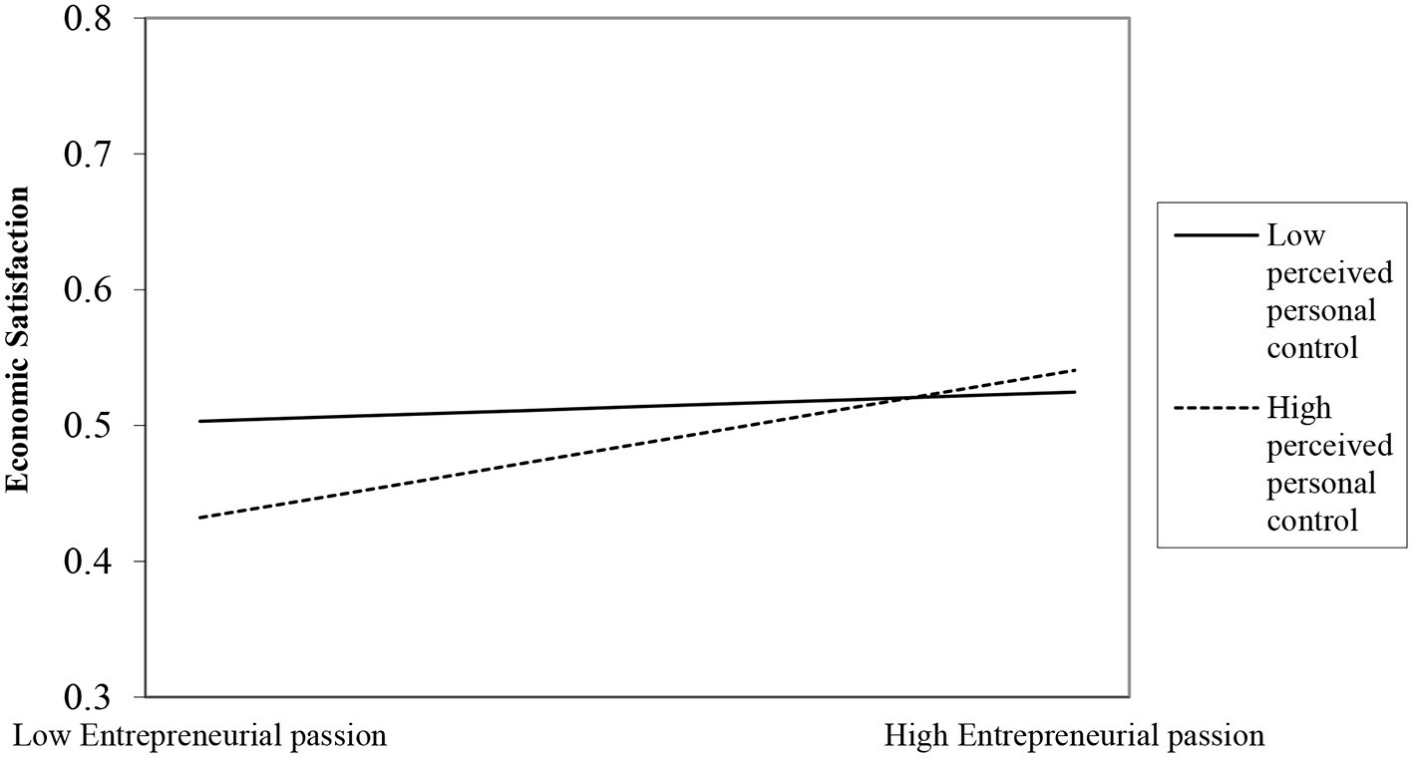
The moderating effect of perceived personal control on entrepreneurial passion and economic satisfaction.

It can be seen from Table 3 that the relationship between the interaction term and the first two result variables is significant; that is, moderation is significant, and thus, hypotheses H4a and H4b have been verified (see Figures 2, 3 for specific moderating effects); the relationship with the last two result variables is not significant; that is, moderation is not significant, and thus, hypotheses H4c and H4d have not been verified.

Further analysis of the significant moderating effect on the spillover to the family domain and that of insignificant to the community domain. We can take a closer look at this empirical test result in terms of the willingness to invest positive resources in the process of entrepreneurs' transitions between roles. Specifically, entrepreneurs tend to spend more energy in their roles in the meso-level combined domain of work-family, where the transition of different roles occurs in a closely connected network. The spillover of entrepreneurs from the work domain into the family domain will require more positive psychological resources, and a greater sense of control can better moderate the relationship between them. However, the meso-level combined domain of the work community requires less energy from entrepreneurs in their roles. In this combined domain, role transition occurs in a loosely connected network, which leads to insignificant moderation.

#### Tests of the spillover-crossover effect

According to the analysis of spillover-crossover effects in the community, this study divides the community into entrepreneurial and non-entrepreneurial communities based on the number of entrepreneurs in the community, which echoes the data of GEM (2019). The specific proportion is 10%; that is, those whose entrepreneurial population accounts for more than 10% (inclusive) are entrepreneurial communities; otherwise, they are non-entrepreneurial communities. It is easy to tell that the level of entrepreneurial passion is higher in entrepreneurial communities than in non-entrepreneurial communities because of the higher proportion of entrepreneurs. The following results (Tables 4–6) are obtained by classifying and testing the growth rates of the entrepreneurial population, the growth rate of the proportion of the entrepreneurial population, and the growth rate of industry agglomeration in the two communities.

**Table 4:** T test results of the growth rate of entrepreneurial population in community.

**Group**	**Obs**	**Mean**	**Std. Err**.	**Std. Dev**.	**[95% Conf. Interval]**	
Non-entrepreneurial community	87	0.7051724	0.1489417	1.389236	0.4090861	1.001259
Entrepreneurial community	152	0.0830395	0.0676411	0.833936	−0.0506058	0.2166848
Combined	239	0.3095063	0.0716978	1.108422	0.1682629	0.4507497
Diff		0.6221329	0.1437542	0.3389336	0.9053322	
Diff = mean (Non-entrepreneurial community)–mean(Entrepreneurial community)						t = 4.3278
Ho:diff = 0						Degrees of freedom = 237
Ha:diff <0		Ha:diff! = 0				Ha:diff > 0
Pr(T < t) = 1.0000		Pr(|T|>|t|) = 0.0000			Pr(T>t) = 0.0000

**Table 5:** *T* test results of the growth rate of the proportion of entrepreneurial population in the community.

**Group**	**Obs**	**Mean**	**Std. Err**.	**Std. Dev**.	**[95% Conf. Interval]**	
Non-entrepreneurial community	87	0.6548069	0.1280922	1.194764	0.4001681	0.9094456
Entrepreneurial community	152	0.0492421	0.0520244	0.6414001	−0.0535477	0.1520319
combined	239	0.2696778	0.0600582	0.9284766	0.1513644	0.3879913
diff		0.6055648	0.1187383		0.3716474	0.8394821
Diff = mean(Non-entrepreneurial community)–mean(Entrepreneurial community)						t = 5.1000
Ho:diff = 0						Degrees of freedom = 237
Ha:diff <0		Ha:diff! = 0				Ha:diff > 0
Pr(T < t) = 1.0000		Pr(|T|>|t|) = 0.0000		Pr(T>t) = 0.0000

**Table 6:** *T* test results of the growth rate of community entrepreneurial industry cluster.

**Group**	**Obs**	**Mean**	**Std. Err**.	**Std. Dev**.	**[95% Conf. Interval]**	
Non-entrepreneurial community	87	1.060486	0.4877133	4.549087	0.0909438	2.030028
Entrepreneurial community	152	0.2475364	0.0845676	1.04262	0.0804478	0.414625
combined	239	0.5434636	0.1865909	2.884625	0.1758829	0.9110442
diff		0.8129493	0.385012		0.0544664	1.571432
Diff = mean (Non-entrepreneurial community)–mean (Entrepreneurial community)						t = 2.1115
Ho:diff = 0						Degrees of freedom = 237
Ha:diff <0		Ha:diff! = 0				Ha:diff > 0
Pr(T < t) = 0.9821		Pr(|T|>|t|) = 0.0358		Pr(T>t) = 0.0179

From the results of Tables 4–6, we can see that among the 239 tracked communities, there are 152 entrepreneurial communities and 87 non-entrepreneurial communities. The *t* tests of the growth rate of the entrepreneurial population, the growth rate of the proportion of the entrepreneurial population, and the growth rate of industry clusters in the two communities are significant at the levels of 0.01, 0.01, and 0.05, respectively, which shows that there are significant differences between entrepreneurial communities and non-entrepreneurial communities. The mean values of the three indicators are all positive, indicating that the effect of community entrepreneurs driving entrepreneurship is positive. That is, entrepreneurial passion is conducive to community-driven entrepreneurship, which is in line with the initial assessment.

However, it is worth noting that the mean values of the growth rate of the entrepreneurial population, the growth rate of the entrepreneurial proportion of the population, and the growth rate of industry clusters in non-entrepreneurial communities are all higher than those in entrepreneurial communities. This finding is inconsistent with the first half of H3 of this study, “The greater the number of community entrepreneurs, the more likely that entrepreneurial passion spillover will drive the development of entrepreneurial clusters in a community.” Because this hypothesis is not only a simple positive correlation but is based on growth indicators such as the growth rate of the entrepreneurial population and the growth rate of the proportion of the entrepreneurial population, it is necessary to make in-depth analyses and judgments to determine why this interesting phenomenon occurs.

Combined with the theoretical inference, emotional contagion is altered by the social comparison mechanism, so we performed a comparative analysis between entrepreneurial operating income (mainly related to entrepreneurial performance) and the per capita income of the community (including the operating income of entrepreneurs and the wage income of non-entrepreneurs), as follows:

Table 7 shows that in 87 non-entrepreneurial communities, the correlation coefficient between the independent variable and the dependent variable is 0.0508, which is positive, and the growth of the proportion of the entrepreneurial population in the representative community has a positive correlation with the difference between the per capita income of the entrepreneurial population and the per capita income of the community. The *p* value is 0.068, which means that the correlation is significant at the level of 0.1, and the R2 is 0.1183.

**Table 7:** The analysis of the correlation between the growth rate of the proportion of entrepreneurial population and the difference between entrepreneurial income and community per capita income in non-entrepreneurial communities.

**Source**	**SS**	**df**	**MS**		**Number of obs** = **87**	
Model	14.5176	3	4.8392		F (3 83) = 3.71	
					Prob > F = 0.0147	
Residual	108.2440	83	1.3041		R-squared = 0.1183	
					Adj R-squared = 0.0864	
Total	122.761673	86	1.4275		Root MSE = 1.1420	
entre_propor_ growthrate	Coef.	Std. Err.	t	P>|t|	[95% Conf.	Interval]
all_population_ 2014	−0.0055	0.0095	−0.58	0.564	−0.0244	0.0134
entre_population_ 2014	−0.2117	0.1173	−1.81	0.075	−0.4449	0.0216
entre_total_ income_ new	0.0508	0.0274	1.85	0.068	−0.0038	0.1053
_cons	1.2793	0.3263	3.92	0	0.6303	1.9283

The difference between the per capita income of entrepreneurs in this community and the per capita income of the community under the condition of a large income gap in the same community, specifically, when the income of entrepreneurs is 1.3 times that of non-entrepreneurs, was analyzed. Table 8 shows that the correlation coefficient between the independent variable and the dependent variable is 0.0678, which is positive, and the growth of the proportion of the entrepreneurial population of the representative community has a positive correlation with the difference between the per capita income of the entrepreneurial population and the per capita income of the community. The *p* value is 0.054, which means that the correlation is significant at the level of 0.1, and the R2 is 0.1652. At the same time, the coefficient between the number of entrepreneurs and the growth rate of the proportion of entrepreneurs is −0.1212, which, to a certain extent, indicates that there is a negative correlation trend between them. It can be considered that entrepreneurial passion's spillover-crossover effect driving entrepreneurship has marginal diminishing utility.

**Table 8:** The analysis of the correlation between the growth rate of the proportion of entrepreneurial population and the difference between entrepreneurial income and community per capita income under the condition of large income.

**Source**	**SS**	**df**	**MS**		**Number of obs** = **58**	
Model	15.3013	3	5.1004		F(3 54) = 3.56	
					Prob > F = 0.0200	
Residual	77.3275	54	1.4320		R-squared = 0.1652	
					Adj R-squared = 0.1188	
Total	92.6288	57	1.6251		Root MSE = 0.1967	
entre_propor_ growthrate	Coef.	Std. Err.	t	P>|t|	[95% Conf.	Interval]
all_population_ 2014	−0.0149	0.0118	−1.27	0.210	−0.0385	0.0087
entre_population_ 2014	−0.1212	0.1496	−0.81	0.421	−0.4212	0.1788
entre_total_ income_new	0.0678	0.0344	1.97	0.054	−0.0011	0.1367
_cons	1.3033	0.4519	2.88	0.006	0.3972	2.2093

Combined with the above analysis, under the condition of a large income gap, the correlation and model stability are more obvious. Therefore, hypothesis H3 proposed in this study has been partially verified. We can draw a modified hypothesis that “The number of entrepreneurs in the community and the income gap jointly predict the spillover-crossover of entrepreneurial passion into entrepreneurial behavior in the community.”

### Robustness check

#### PSM analysis

We chose propensity score matching (PSM) to address endogenous problems (Caliendo and Kopeinig, 2008). Referring to extant research (Binder and Coad, 2013; Hill et al., 2021), we divided entrepreneurs into two groups according to their level of passion and regarded those whose entrepreneurial passion level was higher than 10 as the treatment group and those whose entrepreneurial passion level was lower than 10 (including 10) as the control group. From the treatment group and the control group of entrepreneurial passion to life satisfaction (Table 9), results show that the balance is acceptable. At the same time, the results of the average treatment effect of the treatment group (Table 10) combined with the *p* value controlling heteroscedasticity (ATT = 7.08 > 1.96, *p* = 0.000), the life satisfaction of high entrepreneurial passion spillover is significantly better than that of low entrepreneurial passion spillover, which further verifies Hypothesis 1. Similarly, PSM is used to test for the endogeneity of economic satisfaction, neighbor trust, and donation behavior, and the same results are obtained, which shows that the spillover path of this study is robust.

**Table 9:** Balance test of PSM.

**Variable**	**Matching status**	**Mean value of treatment group**	**Mean value of control group**	**Standard error (%)**	**Standard error reduction**
Gender	U	1.3765	1.3871	−2.2	
	M	1.3773	1.3648	2.6	−18.0
Age	U	42.067	44.68	−25.8	
	M	42.099	41.181	9.1	64.9
Marital status	U	2.0394	2.1129	−11.1	
	M	2.0396	2.0092	4.6	58.7
Education level	U	3.7661	3.1263	34.6	
	M	3.7513	3.7368	0.8	97.7
Family income	U	81277	62713	25.9	
	M	80273	74670	7.8	69.8

**Table 10:** Results of PSM treatment.

**Variable**	**Sample**	**Treatment group**	**Control group**	**Difference**	**S.E**.	**T-stat**
Life satisfaction	Unmatched	3.80289093	3.31989247	0.48299846	0.054627475	8.84
	ATT	3.80145119	3.26297274	0.538478452	0.076025611	7.08
	ATU	3.31989247	3.77491039	0.455017921		
	ATE			0.522033898		

#### Endogeneity test

We used the instrumental variables method to prevent the endogenous problems caused by the mutual causality of entrepreneurial passion, life satisfaction, and economic satisfaction. Based on the practice of Yin et al. (2015), the average level of entrepreneurial passion of other members of the same community is selected as the instrumental variable of this study. Tables 11, 12 show the specific test results of the instrumental variables.

**Table 11:** Instrumental variable testing results (life satisfaction).

**Variable**	**(1) First stage**	**(2) Second stage**
Average entrepreneurial passion of other community members	0.198*** (0.045)	
Entrepreneurial passion		0.330*** (0.101)
Control variables	Controlled
N	1,894	1,894

***P < 0.01; Inside parentheses is the standard error of a coefficient estimate.

**Table 12:** Instrumental variable testing results (economic satisfaction).

**Variable**	**(1) First stage**	**(2) Second stage**
Average entrepreneurial passion of other community members	0.198*** (0.045)	
Entrepreneurial passion		0.274*** (0.106)
Control variables	Controlled
N	1,894	1,894

***P < 0.01; Inside parentheses is the standard error of a coefficient estimate.

Furthermore, the effectiveness and weak identification of instrumental variables are tested. The results of the Durbin-Wu-Hausman test show that there is no problem with weak instrumental variables and reject the original hypothesis that entrepreneurial passion does not exist endogenously at the level of 1.9% (Heckman, 1979; Wooldridge, 2010), indicating that the selected variables are strong instrumental variables. Similarly, in the instrumental variable test to explain economic satisfaction, the same robust test results are obtained. Therefore, the regression results of the instrumental variables show that the positive spillover effect of entrepreneurial passion has a robust causal relationship.

#### Reselect test samples

Referring to the existing research (Naminse and Zhuang, 2018; Miao et al., 2021), for the robustness test, we selected entrepreneurs whose registered residence was an agricultural residence. After screening, there are 1,529 entrepreneurs with agricultural residences in the 1,894 samples of all entrepreneurs. The test results indicate that our study has good robustness.

## Discussion and implications

In this study, we took an enrichment perspective to develop our theoretical model and investigate the positive spillover effect of entrepreneurial passion into the family and community domains. For H1a and H1b, we found that entrepreneurs with a high level of passion are more likely to experience life satisfaction and economic satisfaction in the family domain. Our findings are in line with prior studies (Greenhaus and Powell, 2006; Powell et al., 2019), which argued that positive affect in the work domain would promote high performance in the family domain. Concerning H2a and H2b, the results also confirmed the positive and significant effect of entrepreneurial passion on neighbor trust and donation behavior in the community domain. The results are similar to those reported in existing studies (Aleem and Bano, 2014; Lomas, 2021), suggesting that positive affect has a significant impact on social harmony, including the neighbor relationship and pro-social behavior.

Regarding H3, it was proposed that entrepreneurial passion significantly influences cluster entrepreneurship in a community, which was modified supported. Focusing on entrepreneurial passion, this finding extends the triggering factor of previous research (Nikolaev and Wood, 2018), which implied that there are contagion effects of entrepreneurial activity on self-employment attitudes and choices in the regional dimension. Through the spillover-crossover process, entrepreneurial passion could spread its influence beyond the work domain and impact others in a non-work domain. Communities with a high level of entrepreneurial passion have a higher proportion of entrepreneurs.

Relating to H4, perceived personal control moderates the relationship between entrepreneurial passion and positive outcomes in the family and community domains. Our results indicated that perceived personal control partially moderates the positive spillover of entrepreneurial passion. These are expanded findings in the entrepreneurial context of the extant research (Lang and Heckhausen, 2001; Greenaway et al., 2015), demonstrating that perceived personal control has a positive effect on subjective wellbeing and individual development. Furthermore, our results revealed that the moderating role of perceived personal control is significant for the positive outcomes in the family domain but not in the community domain. Therefore, identifying the boundary conditions of the moderating effects of perceived personal control on the family and community domains could help entrepreneurs gain a deeper and more rational understanding of their entrepreneurial passion's spillovers.

### Theoretical implications

Our research makes four primary contributions to the extant literature. First, our findings contribute to a broader understanding of entrepreneurial passion's boundary of influence in the non-work domains. The prior literature has already conducted relatively rich research on the entrepreneurial performance of entrepreneurial passion in the work domain (Newman et al., 2021; Zhao and Liu, in press). However, most of them have ignored the related effect on entrepreneurs' other embedded environments, such as family and community domains, in the research design. By taking entrepreneurial passion spillover as the key research theme and considering the outcomes in the family and community domains, namely, life satisfaction, economic satisfaction, neighbor trust, donation behavior, and cluster entrepreneurship in the community in the research process, we explore the comprehensive mechanisms that predict positive spillover and crossover effects. Our findings provide novel theoretical insights contributing to the body of knowledge regarding the effects of entrepreneurial passion in the non-work domains.

The second contribution is the use of entrepreneurial passion as an antecedent variable of the work-non-work enrichment theoretical framework. Specifically, literature on enrichment theory has mentioned the instrumental path and affective path for role performance promotion across different domains (Greenhaus and Powell, 2006; Powell et al., 2019). While much of the attention has focused on the conflict of roles in the context of entrepreneurship (Shelton, 2006; Jennings and Mcdougald, 2007; Eddleston and Powell, 2012; Leung et al., 2020), the research on positive mechanisms is still very limited. The investigation of entrepreneurial passion's positive spillover enables us to broaden the research scope of enrichment theory. Entrepreneurial passion is transmitted in the work-family-community mesosystem through the positive emotional resources spiral and the spillover-crossover model, which makes the enrichment mechanism more specific and the measurement of positive balance resources in work-non-work domains more focused. The transfer of affective resources that are derived from passion could help an entrepreneur achieve work-life balance across different domains.

Third, our study illustrates the relevance of perceived personal control in the positive spillover of entrepreneurial passion. Previous studies mainly used perceived personal control as a determinant variable related to personal benefits and social identities (Greenaway et al., 2015; Cichocka et al., 2018), while the interaction effects with other variables are still unclear in the entrepreneurship context. Our study combined perceived personal control with the spillover-crossover model of entrepreneurial passion. The evidence from the moderating test implies that perceived personal control would strengthen the positive relationship between entrepreneurial passion and subjective wellbeing (life satisfaction and economic satisfaction) in the family domain. By examining the moderating role of perceived personal control, we contribute to enhancing the boundary comprehension of entrepreneurial passion spillover theory.

### Practical implications

Our study offers some practical implications for entrepreneurs, family members of entrepreneurs, and community committees. First, it is important for entrepreneurs to recognize that their entrepreneurial passion may help them achieve work-life balance in multiple ways. Entrepreneurs should display their entrepreneurial passion and identities to those in non-work domains with personal control. Entrepreneurs could perceive a higher level of life satisfaction, economic satisfaction, and neighbor trust as a result of positive feedback. Second, family members of entrepreneurs are supposed to give entrepreneurs support for their business and emotional acknowledgment. The positive outcomes brought by the spillover of entrepreneurial passion would facilitate the functioning of the family. Third, it is advisable for community committees to endorse the entrepreneur's passion such that they are more inclined to engage in pro-social activities and share their relevant knowledge with other community members.

### Limitations and future research

Despite our attempts to perform an empirical analysis, due to the limitations of the existing second-hand data, this study still has the following limitations. First, the measurement of entrepreneurial passion is based on the association and causal analysis of the items in the existing scale, and further exploration of its causal relationship can be considered using an experimental design. Second, this study takes samples from a single country. However, situations may vary according to different countries and national cultures, families, and community cultures, especially the comparison before and after COVID-19. Therefore, this study hopes to continue to carry out a cross-border comparison of the spillover effect and its moderating factors of entrepreneurial passion in different cultural situations. Thus, we ask scholars to pay attention to entrepreneurs in different countries and the spillover of entrepreneurial passion into other non-work domains in the future. This will help improve research on entrepreneurs' enrichment within the work-family-community mesosystem.

## Conclusion

Passion is the fuel for entrepreneurial activities, which is crucial for both entrepreneurs and their related actors. There is a lack of positive spillover into the family and community domains. We propose an integrated “work-family-community” framework based on various prior studies. Using the matched samples of entrepreneurs' individuals, families, and communities from the CLDS database, we examine the positive spillover impact. According to the results of our empirical study, entrepreneurs with a greater level of entrepreneurial passion are more likely to assess positively their subjective wellbeing in life and economic aspects. A passion for entrepreneurship would increase the perceived level of trust among neighbors and the likelihood of donations. We hope that future research takes a broader view of entrepreneurship in order to provide a new avenue for treating work-non-work conflict in entrepreneurial life and, as a result, make the world a better place.

## Data availability statement

The raw data supporting the conclusions of this article will be made available by the authors, without undue reservation.

## Ethics statement

This study was carried out in accordance with the recommendations of the Ethical Principles of Psychologists and Code of Conduct of the American Psychological Association (APA). The studies involving human participants were reviewed and approved by the Ethics Committee of the CLDS, Sun Yat-sen University. The participants provided their written informed consent to participate in this study.

## Author contributions

X-HX was in charge of investigation, methodology, writing—original draft, and editing. HF was in charge of conceptualization, methodology, writing—review and editing, and funding acquisition. All authors contributed to the article and approved the submitted version.

## Funding

This work was supported by the National Natural Science Foundation of China (Grant Nos: 72172165; 71772192).

## Conflict of interest

The authors declare that the research was conducted in the absence of any commercial or financial relationships that could be construed as a potential conflict of interest.

## Publisher's note

All claims expressed in this article are solely those of the authors and do not necessarily represent those of their affiliated organizations, or those of the publisher, the editors and the reviewers. Any product that may be evaluated in this article, or claim that may be made by its manufacturer, is not guaranteed or endorsed by the publisher.
